# Nose vs. mouth breathing– acute effect of different breathing regimens on muscular endurance

**DOI:** 10.1186/s13102-024-00840-6

**Published:** 2024-02-09

**Authors:** František Lörinczi, Marián Vanderka, Drahomíra Lörincziová, Mehdi Kushkestani

**Affiliations:** 1 Faculty of Physical Education and Sport, Comenius University in Bratislava, Bratislava, Slovakia; 2 University of Economics in Bratislava, Bratislava, Slovakia; 3 The State University of New Jersey, New Brunswick, USA

**Keywords:** Nasal breathing, Oral breathing, Oronasal Breathing, Bench Press, Repetitions to failure

## Abstract

**Background:**

It has been reported that the way we breathe (whether through the nose or mouth) can influence many aspects of our health and to some extent, sport performance. The purpose of this study was to evaluate the acute effects of different breathing regimens on muscular endurance and physiological variables.

**Methods:**

A randomized experiment to verify the acute effect of different breathing regimens (NN– inhaling and exhaling through the nose; NM– inhaling through the nose, exhaling through the mouth; MM– inhaling and exhaling through the mouth) on the muscular endurance performance was conducted. 107 physically active college students (68 males, 39 females) performed repeated bench press testing protocol (repetitions to failure (RTF) with 60% of body weight for males (BP60), respectively 40% of body weight for females (BP40)) with various breathing regimens (NN, NM, MM) in random order. Heart rate (HR), blood oxygen saturation (SpO2) and perceived exertion by Borg scale (RPE) were measured as well. A short questionnaire, given after the testing protocol and observation during familiarization, was used to detect each subject’s normal breathing approach during resistance training.

**Results:**

In both genders, no significant differences in RTF, RPE and SpO2 were found. No individual case of deviation of arterial oxygen saturation outside the physiological norm was recorded. ​​In the male group, significantly lower HR values were found during the NN trials, compared to during the NM (*p* = 0.033) and MM (*p* = 0.047) trials with no significant differences in females. The HR differences in the males demonstrated a small effect size (NN < NM, d = 0.32; NN < MM, d = 0.30). Questionnaire results suggest that 80% of our participants use NM breathing, 15% use MM breathing and 5% use NN breathing during resistance training.

**Conclusion:**

It seems, that various breathing regimens have none or only minor effect on muscular endurance performance and selected physiological parameters. NN seems to be as efficient as other two regimens, which are mostly used in practice (NM, MM).

## Background

Many medical studies point to the benefits of nasal breathing and negatives of mouth breathing at rest [1, 2]. But still, a significant part of the population are regular mouth breathers [3] or switch to mouth breathing during exercise. Up to this date, a relatively small number of experimental studies address the effect of nasal vs. oral vs. oronasal breathing in the context of physical performance [4]. However, these studies mainly focus on aerobic exercise and none of them focus on resistance training.

### Mouth breathing

Chronic mouth breathing can negatively affect respiratory system and overall health [5, 6]. Inhaling through the mouth introduces unfiltered, poorly humidified air with minimal temperature regulation into the lungs [1], which in turn harms the respiratory system [7]. Night time mouth breathing is connected to a greater incidence of snoring and sleep apnoea [8], while daily mouth breathing gradually induces negative changes in the bone structure and overall facial appearance [1] (e.g.: a narrow face, mouth and nose, higher upper palate, retruded mandible, an elevated position of the hyoid bone, malocclusion, crowded and crooked teeth, secondary halitosis, open bite and dysfunctional jaw joint [2, 9–13]), dental problems (bad breath, dental decay, gum disease) [13], dysfunctions of the facial muscles (mainly around the jaw and lips), trauma to the soft tissues in the airways, enlarged tonsils and adenoids [6, 13, 14], speech problems [15] and a higher prevalence of ADHD [5]. Mouth breathing conducts air mainly to the upper chest, which can be inefficient and tiring [16]. Mouth breathing during physical activity lead to higher loses of water [17] and CO_2_ [18–21], which is associated with a number of negative effects on health, well-being and sport performance [22, 23].

Observational research suggests that more than half of school-aged children are chronic mouth-breathers [24, 25], and 25% of young children have developed sleeping disordered breathing patterns by age six [3].

However, it must be noted that most studies, which point to the negatives of mouth breathing, focus on the chronic and long-term effects of breathing this way habitually [2, 5, 9, 12]. It is less clear, what the health and physiological effects of mouth breathing are when restricted only to the period of exercise training. However, the available evidence suggests that mouth breathing during exercise is associated with the development of exercise induced bronchoconstriction [4].

### Nose breathing

Unlike the oral cavity, the nasal cavities have the function of warming, humidifying, and filtering the inhaled air [26], which contributes to a lower probability of getting colds, flu, allergic reaction, hay fever, or irritable coughing [1]. Nasal breathing is also important for eliminating bronchoconstriction, leading to better prevention and treatment of asthma [27–29]. Nasal breathing helps to form natural dental arches and straight healthy teeth [30]. Nasal breathing better regulates and conditions airflow because of the nose’s intricate structures [31]. Because of the resistance of nasal airways to the airstream, oxygen uptake can be 10–20% higher [32]. Thanks to the negative pressure that must be created, there is a higher activation of the diaphragm and other respiratory muscles [33], which leads to better stabilization of the spine [34, 35] and possible injury-prevention [36]. Nasal breathing contributes to inhalation of increased nitric oxide, which is a potent bronchodilator and vasodilator and has antiviral and antibacterial effects [37, 38] and improves oxygen transport through the body [39].

During rest and light to moderate exercise, pure nasal breathing seems to be sufficient to maintain performance [20]. However, at higher intensities, people switch to oronasal or oral breathing [4]. The ratio of mouth and nose usage can vary among individuals of different races and genders [40]. The cause of the transition stays unclear, although current evidence suggest the switch inbreathing pattern might result from subjective feelings of hypoventilation [41], lower subjective effort [42], or lowering the turbulence associated with airflow through of nasal passage [43]. Available research demonstrates that nasal breathing during steady submaximal exercise, results in a lower respiration rate, a lower ventilation, lower ventilatory equivalent for both oxygen and carbon dioxide, lower oxygen uptake during a given intensity, a lower level of O_2_ and higher CO_2_ in exhaled air [19, 20, 18–21]. Benefits of nasal breathing include a reduction in exercise induced bronchoconstriction, improved ventilatory efficiency, and lower physiological economy for a given level or work [4].. Nasal breathing can reduce achievable maximal oxygen consumption (V˙O_2_max) and peak work in non-adapted individuals [18], but has no significant effect on maximal anaerobic output on similar subjects in a cycling Wingate test [45]. However, in adapted individuals no significant differences in V˙O_2_max or running performance were found [19, 21]. Authors emphasize the fact that individuals can adapt to nasal breathing and higher values end tidal CO_2_ during breathing [4].

The lack of information regarding the acute impact of various breathing regimens (nasal breathing, oral breathing, oronasal breathing) on short-term local muscle endurance performance, in the context of resistance exercise, prompted us to conduct this original study, addressing a gap in the current scientific literature.

## Methods

### Participants

107 physically active individuals (68 males: age: 22.40 ± 1.45 years; body height: 181.07 ± 6.44 cm; body weight: 77.74 ± 9.53 kg; 39 females: age: 21.63 ± 1.60 years; body height: 168.75 ± 5.37 cm; body weight: 63.55 ± 6.38 kg) voluntarily participated in the study. The sample was chosen through a convenience sampling approach, where participants were selected based on their availability and voluntary participation. All participants had experience with resistance training and bench press exercise (intermediate– advanced exercisers). All participants completed 3 repeated measurements in random order for each individual.

### Ethical approval and consent to participate

The study was approved by the ethics commission of the Faculty of Physical Education and Sport, Comenius University in Bratislava (under the number 6/2022) and conformed to the ethical guidelines of the Declaration of Helsinki 2000. All participants provided witnessed oral informed consent prior to entering the study to all authors in the presence of other participants.

### Purpose

The purpose of this study was to evaluate the effects of different breathing regimens (NN– inhaling and exhaling through the nose; NM– inhaling through the nose, exhaling through the mouth; MM– inhaling and exhaling through the mouth) on short term local muscular endurance performance and selected physiological variables in context of resistance exercise.

### Protocol

All procedures were performed at the Faculty of Physical Education and Sport, Comenius University Bratislava, Slovakia. Conditions were kept as stable as possible for repeated measurements (temperature, warm up, time of the day, weight on a barbell, grip width, pace of repetitions).

### Description of the testing protocol

The testing protocol used can be described as repetitions to failure (RTF) on the flat bench press with olympic barbell with resistance– using 60% of body weight for men (BP60) and 40% for women (BP40), an approach which has been validated as an appropriate test to detect muscular endurance of upper body muscles [46, 47]. The weight was adjusted to the nearest 0.5 kg. Grip width and the pace of repetitions was intraindividual (as was natural for participant) but had to be kept the same in all 3 measurements the same. The pace of repetitions was 1011 (around 20–30 repetitions per minute; based on [48, 49]) and grip width was between 1.0 and 1.5 times the biacromial width [50, 51].

### Measurements of physiological variables

Blood oxygen saturation (SpO2), heart rate (HR) and perceived exertion (RPE) were measured right after the test in seated position for 30 s interval. SpO2 was measured by a pulse oxymeter (Viatom Oxymeter PC-60FW), HR by chest strap heart rate monitor (system Polar) and RPE by Borg Scale (6–20). The lowest SpO2 and highest HR were noted.

### Familiarization

All participants were familiarized with the bench press testing protocol and additional measurements (SpO2, HR, RPE) by performing the test with their natural breathing pattern 2 times in separate days. Each participant set suitable grip width (based on set conditions– between 1.0 and 1.5 wider biacromial width and established pace of repetitions (1011). Pace of repetitions was counted by an examiner.

### Testing protocol

All subjects performed 3 experimental repetitions of the bench press testing protocol with randomly selected breathing regimen for each individual (simple randomization). To avoid order effects, the principle of intentional block randomization was used as well to avoid creating significantly different subject numbers within each testing order.

In between test days was at least a 72 h rest period and participants were asked to not have any physical training for at least 24 h before testing. Participants were also asked to not practice any type of training for improving muscular endurance, during the study (between measurements). After 3 repeated measurements, a control retest was conducted with those individuals who had one out of three results of RTF significantly different, to exclude the negative effect of unexpected confounding variables. A persistent significantly different result was evaluated as a criterion for an exclusion.

### Additional measurements

Pre-exercise evaluation was done with a personal scale (MAX MBS2101B) accurate to 100 g.

At the end of the study all participants completed a questionnaire aimed at detecting their breathing preferences and feelings while performing the tests. The questionnaire consisted of two questions: (1) Which type of breathing do you prefer during resistance training? (2) Did you experience the feeling of dried oral cavity? If yes, during which breathing regimen?

### Conditions control

Under the NN condition, participants had medical kinesio tape placed over their mouths in order to prevent any oral breathing. The MM condition was controlled by nose clip, which was placed on the participant’s nose to prevent any nasal breathing. For the NM condition participants were asked to inhale through the nose and exhale through the mouth. Fulfilment of this condition was checked by the examiner during the test.

### Statistical analysis

For statistical analysis, the SPSS (version 25) program was used. The normality of data distribution was verified through the Kolmogorov-Smirnov test and Shapiro-Wilk test. A single factor repeated measures ANOVA was used to detect the significance of differences of results for each dependent variable (RTF, HR, SpO2, RPE) reached under selected breathing conditions (NN, NM, MM). Statistical analysis was done for each gender separately. In line with conventional practices in sports sciences, a significance level (alpha) of 0.05 was employed. Effect size was expressed in significant cases by Cohen’s d [52]. Reliability of the tests used during the testing protocol was assessed from familiarization process by inter-rater intra-class correlation coefficient (ICC), as interpreted by Portney (2009) [53].

## Results

### Repetitions to failure

**Fig. 1:**
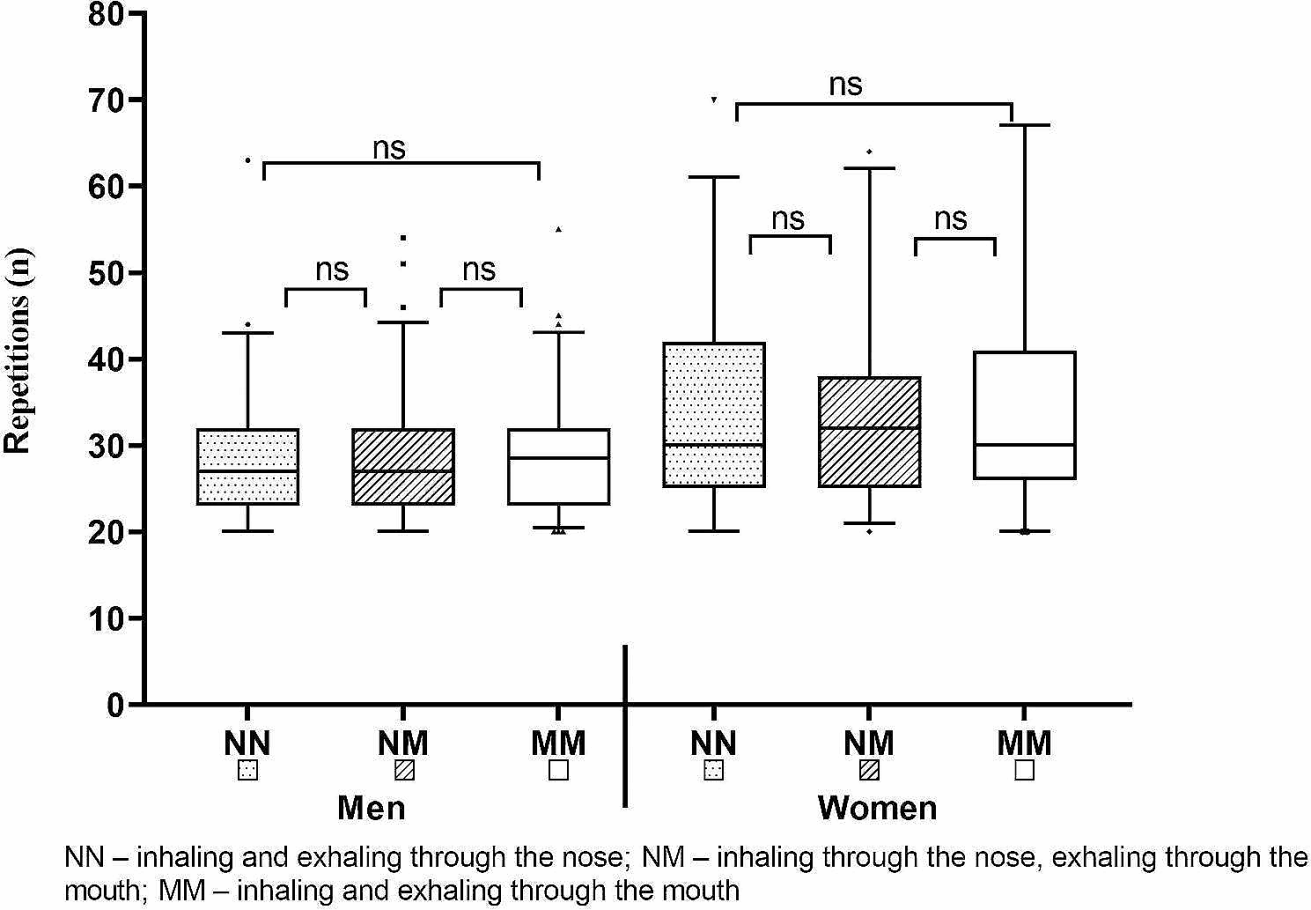
Results and differences in RTF in bench press tests (BP60, BP40) reached by selected breathing regimens (NN, NM, MM)

No significant effect of breathing condition on RTF by gender was found as illustrated in Fig. 1 and summarized below in Table 1. The differences between the mean values ​​were less than 1 repetition on average.

### Heart rate

**Fig. 2:**
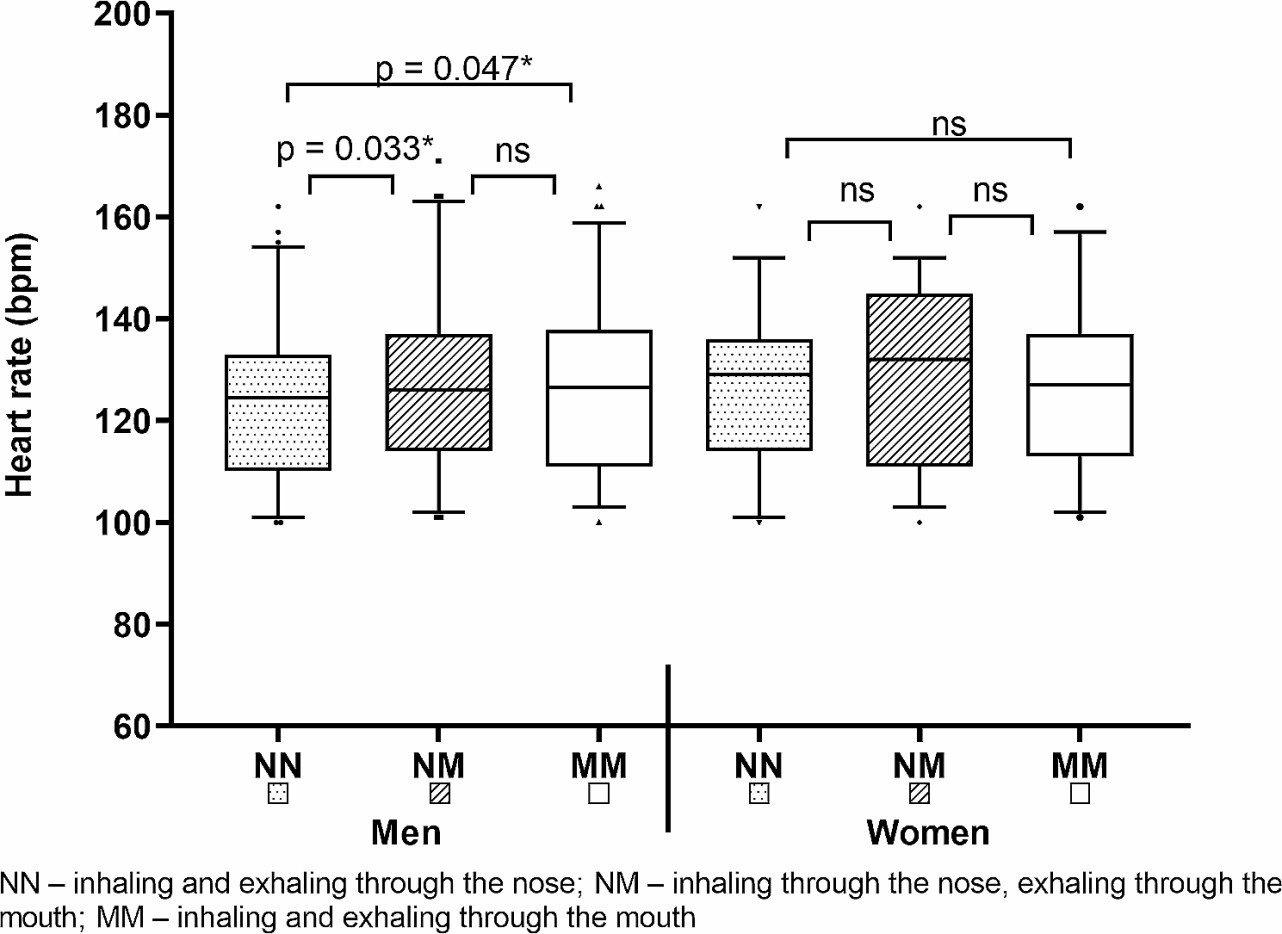
Results and differences in HR after bench press tests (BP60, BP40) reached by selected breathing regimens (NN, NM, MM)

In the male group were found significantly lower HR values ​​after the BP60 with the NN regimen compared to other two regimens that use the mouth (NN < NM, 123.21 ± 15.83 bpm vs. 127.69 ± 16.92 bpm, dif. 4.48 bpm (4%), *p* = 0.047, d = 0.32; NN < MM, 123.21 ± 15.83 bpm vs. 126.97 ± 16.65 bpm, dif. 3.76 bpm (3%), *p* = 0.047, d = 0.30) (see Fig. 2). In the female group no significant differences were found.

### Perceived exertion

**Fig. 3:**
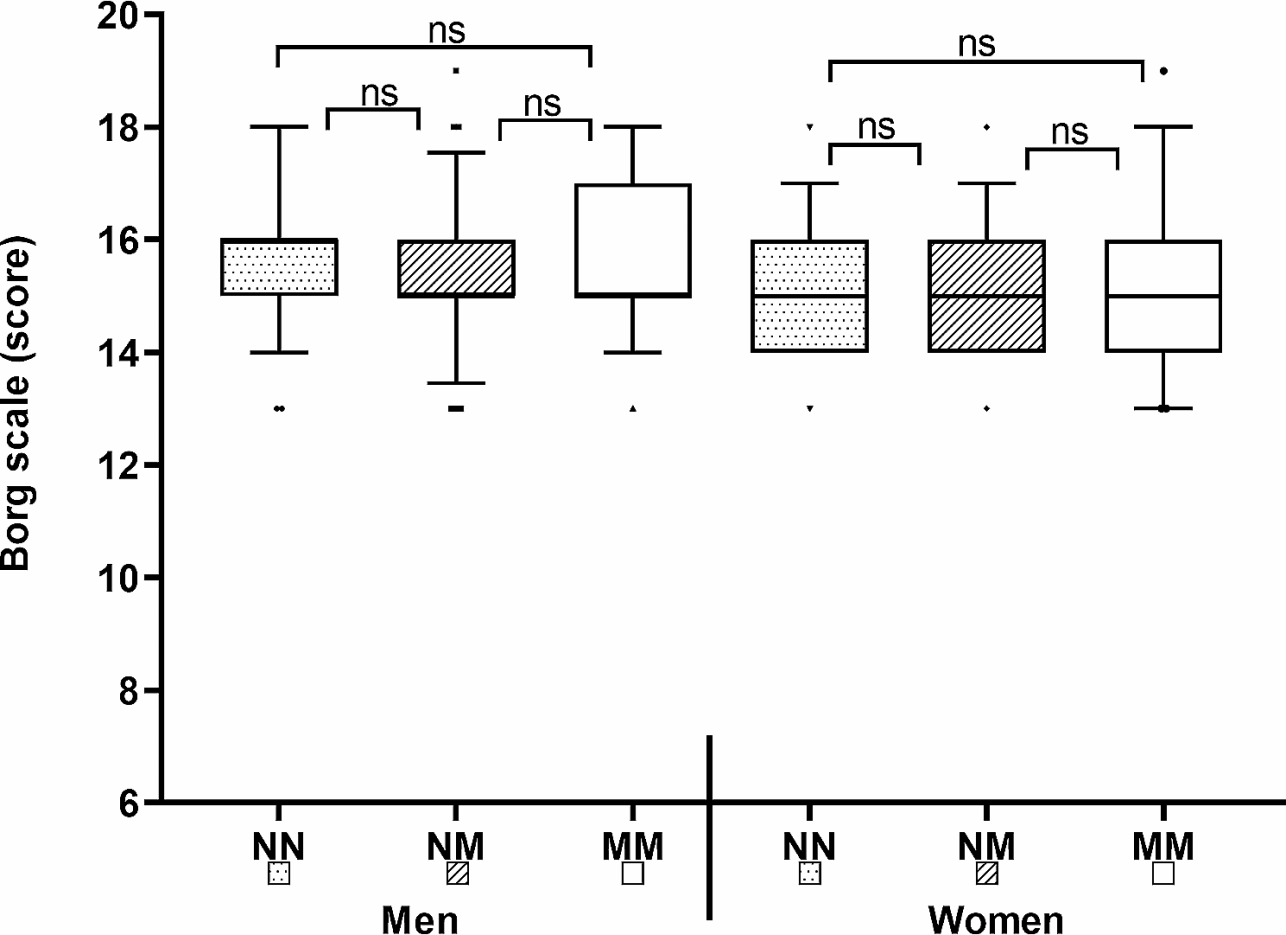
Results and differences in RPE after bench press tests (BP60, BP40) reached by selected breathing regimens (NN, NM, MM)

No significant differences in RPE in both sexes after bench press testing protocols (BP60, BP40) were found (see Fig. 3). The differences between the mean values ​​were less than 1 point of the scale.

### Blood oxygen saturation

During the entire research, no significant drop in SpO2 was recorded. The range of results remained within the physiological norm (95–99%). Average SpO2 values ​​for both sexes were around 98%. No significant differences between various breathing regimens in context of SpO2 were found.

### Summary of results

By conducting a repeated measures ANOVA, no significant differences in RTF, RPE, and SpO2 were observed between different breathing regimens in both sexes (see Table 1). Specifically, for RTF, the analysis revealed no significant differences in men (Wilks’ Lambda = 0.965, F(2,66) = 1.20, *p* = 0.307, η2 = 0.035) or women (Wilks’ Lambda = 0.937, F(2,37) = 1.25, *p* = 0.300, η2 = 0.036).

However, a noteworthy finding emerged in the HR measurements for men. The repeated measures ANOVA showed a significant effect (Wilks’ Lambda = 0.871, F(2,66) = 4.87, *p* = 0.011, η2 = 0.129). Further post-hoc tests revealed that in the male group, the breathing regimen labeled as NN led to significantly lower HR compared to both NM (*p* = 0.033; Cohen’s d = 0.32) and MM (*p* = 0.047; Cohen’s d = 0.30). In contrast, among women, no significant differences were observed in HR (Wilks’ Lambda = 0.998, F(2,37) = 0.04, *p* = 0.962, η2 = 0.002).

For RPE, the repeated measures ANOVA indicated no significant differences in men (Wilks’ Lambda = 0.961, F(2,66) = 1.35, *p* = 0.265, η2 = 0.039) or women (Wilks’ Lambda = 0.988, F(2,37) = 0.224, *p* = 0.800, η2 = 0.012). Similarly, no significant differences were found in SpO2 for men (Wilks’ Lambda = 0.991, F(2,66) = 0.286, *p* = 0.752, η2 = 0.009) or women (Wilks’ Lambda = 0.994, F(2,37) = 0.107, *p* = 0.899, η2 = 0.006).

In summary, the only significant differences were observed in HR within the male group, where NN resulted in a significantly lower HR compared to the other breathing regimens.

**Table 1:** Results in BP60 and BP40 (RTF, HR, RPE, SpO2) between selected breathing regimens (NN, NM, MM)

Group		NN	NM	MM
**RTF (reps; mean ± SD)**				
Men		28.38 ± 7.50	28.66 ± 7.20	28.85 ± 6.84
Women		34.26 ± 12.82	33.79 ± 11.22	34.77 ± 12.89
**HR (bpm; mean ± SD)**				
Men		123.21 ± 15.83	127.69 ± 16.92	126.97 ± 16.65
Women		127.33 ± 15.38	128.05 ± 18.07	127.54 ± 17.14
**RPE (score; mean ± SD)**				
Men		15.57 ± 1.20	15.46 ± 1.26	15.75 ± 1.27
Women		15.41 ± 1.20	15.31 ± 1.24	15.23 ± 1.27
**SpO2 (%; mean ± SD)**				
Men		98.32 ± 0.83	98.22 ± 0.960	98.34 ± 1.06
Women		98.03 ± 1.33	97.92 ± 1.37	97.95 ± 1.28

### Additional measurements

#### Reliability of the tests

BP60 was evaluated with significant (*p* < 0.001) and good reliability (ICC = 0.894). Similarly, reliability of BP40 was significant (*p* < 0.001) and good (ICC = 0.934).

#### Questionnaire

During resistance training, 80% of our participants use NM, 15% use MM and 5% prefer NN. The feeling of dried oral cavity was confirmed in more than half of our respondents (54%), while using MM during the test.

## Discussion

Despite the negative consequences connected with mouth breathing generally [1, 2, 5–17, 28], 95% of the physically active college students enrolled in this study use this way of breathing during resistance training. However, the consequences of mouth breathing, when used exclusively for the period of resistance training, have not been studied directly. Most studies of regular mouth breathing point to long-term and chronic negative effects [2, 5, 9, 12]. It is also not known whether the NM regimen, which is mostly used in practice, is also related to potential health risks. More than half of participants confirmed feeling of dried oral cavity during performing test with MM. This state is probably related to higher water losses and saliva reduction, what can negatively affect dental health [54].

This research points out that it is not necessary to use the mouth for breathing during muscular endurance performance in the range of repetitions used in conventional resistance training, since no significant differences in performance were found. In both genders, the selected breathing regimens (NN, NM, MM) led to similar results in RTF. In the female group, a greater SD of RTF may be related to the more significant heterogeneity of the research sample.

SpO2 and RPE were not significantly affected by breathing conditions as well. The only significant differences were found in context of HR. In the male group, NN led to significantly lower HR, compared to NM (*p* = 0.033; d = 0.32) and MM (*p* = 0.047; d = 0.30). However, effect size revealed only a small effect and in the female group, no differences were found. HR is not the most stable physiological parameter and can be affected by various confounding variables.

In context of SpO2, research shows that it is not possible to reach a state of hypoxemia during resistance exercise to failure, if the exerciser maintains breathing, regardless of the way of breathing (NN, NM, MM). For the future, suitable analytical devices could be used to confirm this statement. For example, the use of near infrared devices e.g. the Moxy muscle oxygen monitor, which measures SpO2 in the muscle, unlike a pulse oxymeter, which measure SpO2 in periphery [55], might provide greater insight into the effect of breathing pattern on oxygen flux during resistive training exercises.

It should be noted that these types of studies require appropriate familiarization, as a significant learning effect in some individuals was found. If individuals lack sufficient experience with resistance training to failure, each try can lead to better results. If this phenomenon persisted after control pretesting, it was evaluated as a criterion for exclusion.

Selected breathing regimens have probably no or only minor effect on endurance type resistance performance and selected physiological variables. Nasal breathing has potential to improve overall health [1, 27], but it is still unknown whether and how it can improve sports performance. In aerobic endurance sports, better breathing efficacy, due to nasal breathing appears to improve physiological economy by improving ventilatory efficiency in subjects previously adapted to breathing this way [4]. However, in context of resistance training, the potential effects of different breathing patterns appear to have no meaningful influence, as suggested by the lack of effect in cyclists during anaerobic Wingate testing [45]. Another line of reasoning suggests that a greater diaphragm activation (due to nasal inhale [33]), could lead to better torso stabilization and potential injury-prevention and performance support [34, 35], but further research is necessary to verify this theory.

This study does not show that any of the breathing regimen is more effective, however it also points out that the most commonly used breathing regimens (NM, MM) are not more effective than a NN regimen, which is potentially healthier and used only minimally. Because of negative associations connected with mouth breathing [1, 2, 5–17, 28],, it is advisable to use nasal breathing whenever it is feasible and as a result act preventively against many undesirable pathological phenomena [1, 2, 8–14].

### Limitation, and suggestion

The primary limitation of this study was the need to measure physiological reposes post exercise, rather than during the exercise testing, due to the type of equipment used and the choice to examine this phenomenon in a field based setting. In so doing more acute physiological effects may have been missed.

## Conclusions

Breathing regimens (NN, NM, MM) have no significant effect on muscular endurance performance and post exercise SpO2 or RPE, with only limited effect on HR.

## Data Availability

The datasets used and/or analysed during the current study are available from the corresponding author on reasonable request.

## Abbreviations

BP40: Flat bench press test with olympic barbell with resistance of 40% of body mass (repetitions to failure)
BP60: Flat bench press test with olympic barbell with resistance of 60% of body mass (repetitions to failure)
HR: Heart rate
MM: Breathing regimen– both inhale and exhale through the mouth (oral breathing)
NM: Breathing regimen– inhale through the nose and exhale the mouth (oro-nasal breathing)
NN: Breathing regimen– both inhale and exhale through the nose (nasal breathing)
ns: Not significant
RPE: Perceived exertion valued by Borg scale (6–20)
RTF: Repetitions to failure
SpO2: Blood oxygen saturation
